# Enhancing Patient Safety Through Predictors of Job Performance in Greek Critical Care Nurses

**DOI:** 10.3390/healthcare13141636

**Published:** 2025-07-08

**Authors:** Thalia Bellali, George Panayiotou, Polyxeni Liamopoulou, Theodora Mantziou, Evgenia Minasidou, Georgios Manomenidis

**Affiliations:** 1 Department of Nursing, International Hellenic University, 57001 Thessaloniki, Greece; tzeniliam@gmail.com (P.L.); dmantziou@gmail.com (T.M.); eminasid68@gmail.com (E.M.); 2 Department of Health Sciences, School of Sciences, European University Cyprus, Nicosia 2404, Cyprus; g.panayiotou@euc.ac.cy; 3 Department of Nursing, Democritus University of Thrace, 68100 Alexandroupoli, Greece; gmanomen@nurs.duth.gr

**Keywords:** patient safety, critical care, nurses, job performance, psychosocial factors

## Abstract

**Background/Objectives**: Job performance among critical care nurses is a pivotal determinant of patient safety. While individual psychosocial factors such as self-care and self-compassion have been separately linked to professional efficacy, limited research has examined their integrated contribution to job performance in high-stakes healthcare environments. **Methods**: A cross-sectional study was conducted in five public hospitals in Northern Greece. A convenience sample of 311 critical care nurses and nurse assistants completed validated self-report measures assessing self-care, self-compassion, mindfulness, physical activity, secondary traumatic stress, and job performance. The data were analyzed using non-parametric statistics and multivariate linear regression. **Results**: Higher levels of self-care (*p* = 0.003) and self-compassion (*p* = 0.042), and lower levels of secondary traumatic stress (*p* = 0.04), were significantly associated with better job performance. The final regression model explained 31% of the variance in performance scores (R^2^ = 0.31). Mindfulness and physical activity were not significantly associated with job performance. Secondary traumatic stress emerged as the strongest negative predictor. **Conclusions**: Internal psychosocial resources, particularly self-care and self-compassion, significantly contribute to job performance among critical care nursing personnel. These findings underscore the relevance of embedding staff well-being strategies into organizational patient safety agendas. This multidimensional model provides a novel framework for developing targeted interventions in high-acuity healthcare settings.

## 1. Introduction

Ensuring patient safety in critical care settings remains a top global priority, especially given the high demands, complex interventions, and emotional toll of intensive care environments. Among the various systemic and clinical strategies proposed, an emerging body of research highlights the central role of frontline healthcare professionals’ well-being in preventing errors and promoting optimal outcomes [1,2]. Despite this, studies specifically targeting predictors of job performance among critical care nurses and their indirect impact on patient safety are still limited.

Patient safety is a structured system of organized activities that fosters cultures, processes, procedures, behaviors, technologies, and environments in healthcare, aimed at continuously and sustainably mitigating risks, decreasing the incidence of preventable damage, minimizing the likelihood of errors, and lessening the effect of harm when it occurs [3]. Patient safety is paramount in critical care settings, such as Intensive Care Units (ICUs) and Emergency Departments (ERs), due to critically ill patients’ medical status, acuity, and vulnerability [4]. Rapid decision making and the need for life-sustaining interventions elevate the potential for clinical errors, which remain one of the most serious threats to patient safety in critical care settings [5].

Increased incidences of clinical mistakes, particularly in high-pressure environments, have been directly linked to factors such as burnout [6], fatigue [7], and poor mental health among nurses [8]. These factors collectively contribute to the degradation of their performance, thereby jeopardizing patient safety. However, several protective psychosocial and behavioral factors have been acknowledged to enhance the performance of nurses, thus contributing significantly to nurses’ ability to make sound clinical decisions. Emotional resilience, mindfulness, physical self-care, and psychological safety are recognized as foundational competencies that influence both care quality and safety cultures [9].

Emotional intelligence (EI) is defined as “the ability to perceive and manage emotions of oneself and others” [10]. It is essential for stress management, enhancing well-being, maintaining composure during emergencies, and improving clinical decision-making, which reduces the likelihood of errors [11]. Studies have shown that high levels of EI among nurses are associated with improved clinical competence and clinical judgement [12], more effective interaction with patients [13], and enhanced cooperation in multidisciplinary teams [14]. These qualities foster a safety culture by promoting clear communication and early identification of potential risks, thus ensuring the safety of care delivery.

Mindfulness is another protective factor that enhances nurses’ focus, reduces cognitive errors, and supports emotional regulation in high-stress environments [15]. Mindfulness is the ability to remain fully present and aware of one’s thoughts, emotions, and surroundings nonjudgmentally and without criticism [16]. Research suggests that mindful nurses are more attentive to their patients [17] and are more likely to adhere to safety protocols, resulting in a higher level of patient safety [18].

For nurses working in high-intensity environments such as critical care units, self-compassion is recognized as a vital psychological resource. It refers to being supportive, understanding, and kind toward oneself during times of failure, inadequacy, or personal suffering [19]. Self-compassion can buffer the adverse effects of stress and mitigate the harsh self-criticism that has been shown to impair cognitive function and decision-making, both of which are important for maintaining patient safety [20]. Research indicates that nurses who practice self-compassion are more likely to acknowledge clinical mistakes without fear, learn from them, and ultimately improve patient safety [21].

Secondary traumatic stress is often described as emotional and physical exhaustion resulting from prolonged exposure to patient suffering [22]. When it occurs, nurses may display reduced empathy towards patients, emotional numbness, and decreased job satisfaction [23]. This limited emotional engagement can impair clinical judgement and increase the possibility of errors [24]. Studies show that secondary traumatic stress affects the quality of nursing care and is strongly associated with reduced adherence to safety protocols [25,26].

The role of physical activity as a key factor in supporting the health, cognitive function, and overall well-being of nurses, which are factors that indirectly influence nurses’ ability to remain alert and make sound clinical decisions, thus enhancing the maintenance of patient safety, has been suggested by various research [27,28,29]. Physically active nurses also report lower burnout and fatigue, which contribute to errors and reduced mental clarity and safety [30,31].

Finally, nurses’ self-care is recognized as essential in promoting patient safety. Engaging in self-care helps nurses create a healing environment that alleviates the risks of burnout and secondary traumatic stress while enhancing patient satisfaction [32,33]. In addition, self-care supports better decision-making by enabling nurses to respond more effectively to high-pressure situations and patients’ complex needs [34].

Given critical care nurses’ well-documented challenges—including emotional exhaustion, secondary traumatic stress, and cognitive overload—there is a growing interest in identifying protective personal factors that may enhance professional functioning and contribute to safer care delivery. While previous studies have explored elements such as mindfulness, resilience, or burnout in isolation, few have examined their combined predictive value in the context of job performance. The present study aimed to investigate the extent to which individual-level psychosocial and behavioral factors—such as self-care, self-compassion, physical activity, mindfulness, and secondary traumatic stress—predict job performance among Greek nursing personnel in critical care settings.

To our knowledge, this is the first study to simultaneously investigate the roles of all these factors within an integrated predictive model of job performance in critical care nursing staff. This multidimensional approach provides a novel perspective on how individual-level resources may buffer the demands of high-intensity healthcare environments and support institutional patient safety goals. By examining these predictors within the high-stakes environment of ICUs and ERs, the study aimed to elucidate their indirect yet pivotal role in enhancing patient safety, recognizing that optimal nurse performance is a cornerstone of error prevention, clinical responsiveness, and high-reliability care delivery. The purpose of this study was to investigate the relationship between self-care, self-compassion, secondary traumatic stress, mindfulness, and physical activity, and their predictive value for job performance among ICU and ER nursing personnel. We hypothesized that higher levels of self-care, self-compassion, mindfulness, and physical activity, and lower levels of secondary traumatic stress, would be significantly associated with better job performance.

## 2. Materials and Methods

### 2.1. Study Design and Setting

A cross-sectional descriptive study was conducted in critical care settings (ICU and ER) of 5 secondary and tertiary public hospitals in Northern Greece between September 2024 and December 2024. The study’s description was disseminated to potential participants via multiple channels, including informational flyers and posts placed in high-traffic areas of hospitals, such as break rooms, elevators, notice boards, and staff portals. Participation was entirely voluntary, and no incentives were offered. To ensure that participation was free from undue influence, especially from supervisors or hospital administrators, the recruitment process was conducted independently by the research team and no managerial staff were involved in recruitment. Interested personnel contacted the first author and received further information about the study. After providing participants with an overview of the research procedures, participants received the study link.

### 2.2. Data Collection

A convenience sample of nursing personnel working in critical care settings was considered eligible for the study. The study targeted nurses who met the following criteria: nurses who had permanent employment status, at least one year of working experience, and involvement in direct care of patients in the ICU and ER departments. Nursing personnel in non-clinical roles, such as administrative staff and supervisors, were excluded from participation. Data were collected using a self-administered questionnaire distributed through Google Forms. Completion time was approximately 10–15 min.

### 2.3. Ethical Considerations

The study was conducted according to the Declaration of Helsinki, and the protocol was approved by the scientific committee of all the hospitals involved. At all stages of the study, participants were informed that their involvement was voluntary and anonymous and that they could withdraw at any time without consequence.

### 2.4. Data Collection Tools

A structured questionnaire was used with the following: (a) demographic and occupational characteristics, (b) the Self-Care Scale, (c) the Self-compassion scale (SCS), (d) the International Physical Activity Questionnaire (IPAQ), (e) the Mindful Attention and Awareness Scale (MAAS), (f) the Secondary Traumatic Stress subscale (STS) of The Professional Quality of Life Scale (ProQuol), and (g) the Job Performance Scale (JPS).

### 2.5. Demographic and Occupational Characteristics

The demographic and occupational characteristics questionnaire was constructed for the study. It included the following questions: gender, age, marital status, level of education, years of experience, and working department.

### 2.6. The Self-Care Scale

The Self-Care Scale was developed based on existing literature [35,36] and includes 35 items designed to assess the self-care practices of individuals. This scale has previously been utilized in healthcare professionals (HPs) in Greece [37] and demonstrated excellent internal consistency (Cronbach’s α > 0.90). The response options ranged from 1 (never) to 6 (always), with higher scores reflecting higher levels of self-care. The total score on the self-care scale ranges from 35 to 210. The Cronbach’s alpha internal consistency for the study was 0.926, indicating an excellent level of reliability.

### 2.7. The Self-Compassion Scale

The Self-Compassion Scale (SCS) examines the relationship between self-compassion, positive psychological health, and the Five-Factor Model of Personality [38]. Self-compassion involves being kind to oneself in times of pain or failure, perceiving one’s experiences as part of a larger human experience, and keeping painful thoughts and feelings in balance. The SCS consists of 26 items that are rated on a 5-point Likert scale, ranging from “Rarely (1)” to “Almost Always (5)”, with the total score calculated as the mean of the responses. Scores on the scale from 1 to 2.5 indicate low self-compassion, 2.5 to 3.5 indicate moderate self-compassion, and 3.5 to 5.0 indicate high self-compassion [38]. The SCS has been translated into Greek, and it is an effective tool for measuring self-compassion within the Greek cultural context, with adequate psychometric properties [39]. The Cronbach’s α for the study was 0.866, indicating good reliability.

### 2.8. The International Physical Activity Questionnaire

The International Physical Activity Questionnaire (IPAQ) [40] is a widely used tool for assessing participants’ physical activity (PA). The original version consists of 31 questions, while the short form includes 7. Participants are asked to record the frequency and duration of their involvement in activities of varying intensity over the past seven days. The IPAQ calculates PA in terms of MET-minutes· week^1^ for walking, moderate-intensity, and vigorous-intensity activities. The Metabolic Equivalent of Task (MET) represents the ratio of the work metabolic rate to the resting metabolic rate [41]. Based on the total MET-minutes·week^1^, PA levels are categorized as low (Total PA score < 600 MET-min·week^−1^), moderate (Vigorous PA score ≥ 480 MET-min·week^−1^ or Total PA score ≥ 600 MET-min·week^−1^), or high (Vigorous PA score ≥ 1500 MET-min·week^−1^ or Total PA score ≥ 3000 MET-min·week^−1^). The IPAQ has been validated for the Greek population [42].

### 2.9. The Mindful Attention Awareness Scale

The Mindful Attention Awareness Scale (MAAS) [43] consists of 15 questions that measure an individual’s tendency to operate on “autopilot”, without focusing attention on the present experience. Responses are given on a 6-point Likert scale, ranging from “Almost Always (1)” to “Rarely (6)”. According to the authors, the scale does not include other elements of mindfulness, such as non-judgmental behavior, as awareness and attention to the present moment are considered the most fundamental components of this concept. The scale has been validated in Greek in a student sample [39]. The Cronbach’s α for the study was 0.894, indicating good reliability.

### 2.10. The Secondary Traumatic Stress Scale

The Secondary traumatic stress Scale from the Professional Quality of Life Scale (ProQuoL) [44] assessed secondary traumatic stress. The Secondary traumatic stress Scale consists of 10 items evaluating compassion fatigue/secondary traumatic stress, and responses are rated on a 6-point Likert scale ranging from “Never (1)” to “Very Often” (6) [44]. The ProQOL questionnaire has been translated and validated in Greek and has been used in Greek nurses [45]. The Cronbach’s α for the study was 0.759, indicating acceptable reliability.

### 2.11. The Job Performance Scale

The Job Performance Scale (JPS) (adapted from Goodman and Svyantek’s scale) [46] consists of 6 statements: three statements assess in-role performance (e.g., the statement “You meet performance criteria”), while the remaining three statements evaluate extra-role performance (e.g., “You willingly take on tasks that are not part of your duties but are beneficial to the overall image of the organization”). Participants’ responses are rated on a Likert scale ranging from “Does not describe me at all (0) “ to “Describes me perfectly (6)”. It has been adapted into Greek [47], and the Cronbach’s α for the overall study’s instrument was 0.885, indicating good reliability.

### 2.12. Data Analysis

Data analyses used the Statistical Package for Social Sciences, version 26 (Armonk, NY, USA, IBM Corporation). The normality of the distribution of observed numeric variables was assessed using the Kolmogorov–Smirnov test. Categorical variables are presented as numbers and percentages, while continuous variables are expressed as mean ± standard deviation. Since the regularity check showed no normal distribution of variables, non-parametric methods were conducted. Differences in job performance based on gender, marital status, level of education, and working department were examined using the Mann–Whitney Z-test and the Kruskal–Wallis H-test. Spearman’s correlation coefficient was performed between all other independent variables and job performance. Variables showing a significant association with job performance in the bivariate analysis were entered into a multiple regression model as predictors, with job performance as the dependent variable.

## 3. Results

A total of 311 questionnaires were received. The respondents included 219 (70.4%) nurses and 92 (29.6%) nurse assistants. The sample consisted mainly of females (72.3%), who lived with their family (60.8%) and worked in the ICU (59.2%). The demographic and occupational characteristics of the participants are presented in Table 1.

The descriptive statistics for the six scales (Self-care, IPAQ, SCS, MAAS, CFS, and JP) are presented in Table 2. The mean score for the Self-care scale was 140.6 (±25.76), indicating a high level of self-care. The mean score for the MAAS was 4.22 (±0.82) above the mid-point, suggesting a high level of mindfulness, while the mean score for the SCS was 2.95 (±0.54), indicating moderate self-compassion. The mean score for the CFS was 24.24 (±5.68), indicating (total score on the CFS ranges from 10 to 60) low levels of secondary traumatic stress. The nursing personnel’s total PA was 1592.9 (±743.8) met·min·wk^−1^, indicating a moderate PA profile classification (PA class 1.78 ± 0.7).

The bivariate analysis between the independent variables and JP is shown in Table 3. There was a significant difference between JP and gender (female mean = 45.23 and male mean = 53.76) (*p* = 0.002), greater age (rho = 0.558, *p* = 0.041), nursing personnel (nurses mean = 49.23 and nurse assistants mean = 45.36), and greater working experience (rho = 0.419, *p* = 0.021). Moreover, there was a positive association between JP and the level of self-care (higher) (*p* = 0.018) and self-compassion (higher) (*p* = 0.014), while there was a negative association with secondary traumatic stress (*p* = 0.028).

The multivariate linear regression showed that the educational level of nursing personnel, self-care, self-compassion, and lower levels of secondary traumatic stress were significant predictors of higher job performance. Secondary traumatic stress emerged as the strongest predictor. The model was statistically significant (F = 3.873, *p* = 0.019, R^2^ = 0.31, adjusted R^2^ = 0.26), explaining 31% of the variance in job performance. Multicollinearity was assessed using variance inflation factors (VIFs), with all values ranging between 1.12 and 1.89, indicating no serious multicollinearity among predictors. Residual analysis, including a visual inspection of Q-Q plots and residual vs. fitted value plots, confirmed the approximate normality and homoscedasticity of the residuals, suggesting that the assumptions of linear regression were met. Variables such as mindfulness and physical activity (IPAQ) were excluded from the regression model due to their non-significant associations with job performance in the bivariate analysis. These findings are presented in Table 4.

## 4. Discussion

This study investigated the influence of individual-level psychosocial and behavioral factors—namely self-care, self-compassion, physical activity, mindfulness, and secondary traumatic stress—on job performance among nurses and nurse assistants in critical care settings. The results revealed that higher levels of self-care and self-compassion, alongside lower levels of secondary traumatic stress, were significant predictors of improved job performance. These findings align with emerging healthcare models that view staff well-being not as peripheral, but as central to patient safety and high-reliability care systems [48,49,50].

Self-care emerged as a significant positive predictor of job performance. Previous studies have highlighted that nurses who actively engage in self-care practices report higher professional satisfaction, better psychological well-being, and reduced burnout—all contributing to improved performance and safer clinical practices [51,52]. Moreover, self-care practices have been associated with enhanced resilience and coping mechanisms, which are essential in high-stress environments like critical care units [24,52].

Similarly, self-compassion significantly contributed to job performance. Nurses with higher self-compassion tend to exhibit greater emotional regulation and psychological resilience, mitigating the impact of workplace stress and secondary traumatic stress [53]. One plausible explanation for the beneficial effect of self-compassion on job performance lies in its role as a self-regulatory mechanism. Self-compassionate individuals are more likely to engage in adaptive emotion regulation strategies, such as cognitive reappraisal and self-soothing, which enable them to recover more effectively from clinical errors or emotionally challenging patient interactions [54]. This emotional flexibility may help preserve mental bandwidth for task-focused behavior, enhance interpersonal communication, and reduce avoidance-based coping, which are essential factors in maintaining high-quality performance in critical care environments. Moreover, self-compassion has been associated with lower levels of rumination and self-criticism, which could otherwise impair decision-making accuracy and attentional control in high-stakes settings [24,55].

Our findings revealed that higher levels of self-care and self-compassion, along with lower levels of secondary traumatic stress, were significantly associated with better job performance (*p* < 0.05), which is in turn a documented protective factor against clinical errors [46,56]. These findings align with global research trends among critical care nurses. For instance, in a mixed-methods study design in acute medical care hospital nurses, the use of self-compassion as a coping strategy was reported as beneficial for the caregiver, with corresponding benefits for the patient needing care [57]. Also, in a cross-sectional study in Turkey, nurses reported moderate levels of self-compassion, which significantly predicted caring behavior [58]. Given that enhanced nurse performance correlates directly with safer patient care and reduced adverse events [59,60], our results underscore the necessity of supporting personal wellness strategies in critical care professionals as a core patient safety intervention.

However, in contrast, secondary traumatic stress was negatively associated with job performance, echoing findings from past research highlighting its adverse impact on concentration, clinical decision-making, and interpersonal functioning [49,61]. Elevated levels of secondary trauma and secondary traumatic stress are known to impair recognition of subtle clinical cues, increase irritability, and promote disengagement from patients, representing factors that are all detrimental to patient safety [61,62].

Although mindfulness was not statistically significant in the multivariate model, prior studies have documented its positive effects on nurse well-being and professional efficacy. Mindfulness-based interventions (MBIs) have been shown to reduce burnout, enhance compassion satisfaction, and foster attentional control, which are particularly valuable traits in the cognitively demanding context of critical care [55,63]. Likewise, regular physical activity has been associated with decreased stress and improved emotional regulation among nurses [52,64]. However, contrary to previous studies that reported significant associations between physical activity through self-care and improved job performance [51,52,65], the present study did not identify a statistically significant relationship between IPAQ scores and performance indicators. The absence of a significant association between physical activity and job performance in this study may be explained by several contextual and methodological factors. First, the relatively low variability in the IPAQ scores—predominantly in the low-to-moderate activity range—may have limited the ability to detect meaningful effects. Second, systemic constraints in the Greek public healthcare setting, including irregular shift patterns, staffing shortages, and insufficient institutional support for wellness initiatives, may reduce the feasibility and impact of sustained physical activity among nursing staff. Furthermore, it is plausible that any beneficial effects of physical activity on work performance are indirectly mediated through mechanisms such as improved sleep quality, emotional regulation, or reduced fatigue—variables not directly captured in the current model. In terms of model specification, both mindfulness and physical activity were excluded from the final regression due to their non-significant associations with job performance in the bivariate analysis. It is also possible that collinearity with variables such as self-care or self-compassion influenced their predictive ability. These considerations underscore the importance of future research aimed at disentangling the complex pathways through which physical activity may contribute to professional functioning in high-stress clinical environments.

All the above findings underscore the importance of integrating psychological wellness into institutional patient safety strategies. The safety of critically ill patients depends on adherence to clinical guidelines and the cognitive, emotional, and physical functioning of the nurses delivering care [48,52]. Embedding structured wellness programs—including self-care workshops, mindfulness training, and trauma-informed peer support—into continuing professional development may contribute significantly to a safer care environment [51].

This study has some limitations. Its cross-sectional design precludes causal inferences. The reliance on self-reported measures raises the possibility of response bias due to social desirability. The number of nurses who declined to participate and the total number of employed nurses during the study period were not recorded. This limits our ability to determine the response rate and assess the representativeness of the sample. Also, an a priori power analysis was not conducted to determine the required sample size, which limits our ability to assess whether the study was adequately powered to detect small-to-moderate effects. Additionally, the exclusion of mindfulness and physical activity from the regression model—despite theoretical relevance—represents a methodological limitation. These variables were omitted due to their non-significant bivariate associations with job performance and potential conceptual overlap with the included predictors. Furthermore, the sample was geographically limited to hospitals in Northern Greece, which may limit its generalizability to other regions or healthcare systems. Despite these limitations, the study has several methodological strengths. It employed validated and widely used instruments to measure key psychosocial variables, enhancing the reliability and comparability of results. Additionally, the sample size was adequate for multivariate analyses and included participants from multiple hospitals, improving the ecological validity of the findings within critical settings. Nonetheless, the study provides valuable insights into modifiable predictors of job performance, with practical implications for workforce development and patient safety initiatives. Although the sample was contextually situated in the Greek healthcare system, the combination of individual-level variables examined has not been widely investigated in an integrated manner in the international literature. This multidimensional approach offers a novel perspective that can inform future research and policy across diverse healthcare contexts, especially as these psychosocial constructs are considered universally relevant to nursing performance and resilience in critical care settings.

However, we acknowledge that institutional structures, staffing models, and cultural norms regarding mental health and self-care vary significantly across countries. These differences may influence both the expression of these psychosocial factors and their impact on job performance. As such, the generalizability of our findings to other healthcare systems should be approached with caution. Replication studies in other national and institutional contexts are needed to assess the robustness of these associations across settings.

Future research should explore longitudinal or intervention-based designs to evaluate causal pathways between staff wellness and patient safety indicators, such as adverse event rates or error reporting frequency. Examining the moderating role of organizational culture, leadership style, and team cohesion may offer a more comprehensive understanding of the mechanisms linking individual well-being to patient outcomes in critical care.

Given the established link between self-compassion, self-care, and job performance, hospital leadership and policymakers should consider integrating structured psychosocial support into staff development programs. Interventions such as self-compassion training, resilience workshops, and trauma-informed supervision may not only enhance staff well-being but also serve as proactive strategies for minimizing latent threats to patient safety [53,66]. Embedding such initiatives into continuing education curricula and accreditation standards could institutionalize a culture of emotional competence, clinical vigilance, and safer care delivery in critical care settings.

## 5. Conclusions

The present study points to the relevance of internal psychosocial resources in sustaining nursing performance within critical care environments. In line with our hypothesis, self-care and self-compassion emerged as significant positive predictors of job performance, while secondary traumatic stress was a significant negative predictor. These findings underscore the importance of internal psychological resources in maintaining optimal functioning under the demanding conditions of ICU and ER settings. Although mindfulness and physical activity were not significant in the final model, their known indirect benefits suggest value for future exploitation. Beyond identifying key associations, this study calls attention to the systemic value of embedding well-being-oriented frameworks into patient safety agendas. Supporting frontline professionals through tailored organizational interventions and integration of self-compassion training in continuing education programs may serve as a workforce resilience strategy and a pathway to safer clinical care. This multidimensional model offers a valuable blueprint for designing integrated workforce development and patient safety strategies in high-acuity healthcare settings.

## Figures and Tables

**Table 1:** Demographic and occupational characteristics.

Variables	n (%)
Gender	
*Female*	225 (72.3)
*Male*	86 (27.7)
Age ^a^	46.23 (7.4)
Marital status	
*Live with my partner*	83 (26.7)
*Live alone*	39 (12.5)
*Live with my family*	189 (60.8)
Nursing personnel	
*Nurses*	219 (70.4)
*Nurse assistants*	92 (29.6)
Working experience ^a^	17.4 (9)
Department	
*ICU*	184 (59.2)
*ER*	127 (40.8)

^a^ Mean (SD).

**Table 2:** Descriptive statistics for Self-care scale, SCS, MAAS, CFS, JP, and IPAQ.

Scale	Mean	Standard Deviation
Self-care scale	140.6	25.76
SCS	2.95	0.54
MAAS	4.22	0.82
CFS	24.24	5.68
JP total	27.53	5.90
In-role performance	13.61	3.14
Extra-role performance	13.91	3.20
IPAQ total (MET·min·wk^−1^)	1592.9	743.8
PA Classification	1.78	0.7

**Table 3:** Summary table of correlations between independent variables and JP.

Independent Variable	Test Used	Test Statistic (Z/H/ρ)	JP *p*-Value
Gender	Mann–Whitney U	1.860	0.002 *
Age	Spearman’s ρ	0.558	0.041 *
Living arrangement	Kruskal–Wallis H	0.925	0.630
Nursing personnel	Mann–Whitney U	1.142	0.025 *
Working experience	Spearman’s ρ	0.419	0.021 *
Department	Mann–Whitney U	−0.459	0.145
Self-care Scale	Spearman’s ρ	0.479	0.018 *
SCS	Spearman’s ρ	0.569	0.014 *
MAAS	Spearman’s ρ	0.581	0.275
CFS	Spearman’s ρ	−0.474	0.028 *
IPAQ-total	Spearman’s ρ	0.623	0.451

* Significant at *p* < 0.05 level.

**Table 4:** Summary of multiple regression model with job performance as dependent variable.

Variables	B	Standard Error	*t*	*p*	95% CI
Gender	−0.036	0.772	0.047	0.963	−1.547, 1.481
Age	0.057	0.061	0.932	0.352	−0.063, 0.177
Educational level of nursing personnel	0.642	0.306	2.10	0.038 *	0.042, 1.242
Working experience	0.002	0.051	0.046	0.963	−0.103, 0.098
Self-care Scale	0.152	0.048	0.58	0.003 *	0.130, 0.181
SCS	0.132	0.033	2.717	0.042 *	0.063, 0.203
CFS	−0.081	0.042	2.856	0.04 *	−0.164, 0.003

F = 3.873, *p* = 0.019, R^2^ = 0.31, * *p* < 0.05.

## Data Availability

The data that support the findings of this study are available from the corresponding author upon reasonable request. The data are not publicly available due to institutional and ethical restrictions related to participant confidentiality.

## Abbreviations

The following abbreviations are used in this manuscript:

ICU	Intensive Care Unit
ER	Emergency Department
SCS	Self-Compassion Scale
IPAQ	International Physical Activity Questionnaire
MAAS	Mindful Attention and Awareness Scale
STS	Secondary Traumatic Stress
ProQuol	Professional Quality of Life Scale
JPS	Job Performance Scale
PA	Physical Activity
